# Development of a hamster model of spontaneous hypertriglyceridemia in diabetes

**DOI:** 10.1002/ame2.12490

**Published:** 2024-11-12

**Authors:** Ya‐Hong Ma, Yuhui Wang, George Liu

**Affiliations:** ^1^ Department of Endocrinology Beijing Puren Hospital Beijing China; ^2^ Institute of Cardiovascular Sciences Peking University Health Science Center Beijing China; ^3^ Yohan Bioteck Co. Hangzhou China

**Keywords:** diabetes, hamster, hypertriglyceridemia, LDL receptors

## Abstract

Hypertriglyceridemia (HTG) often accompanies diabetes and is considered a risk factor for diabetic vascular complications. However, inducing diabetic HTG typically requires high‐fat diets in certain animal models. Leveraging our newly developed LDL receptor knockout hamster model, which exhibits features akin to human lipid metabolism, we sought to determine whether these animals would develop HTG without dietary manipulations in diabetes. Diabetes was induced via intraperitoneal injection of STZ in wild type and heterozygous LDL receptor deficient hamsters. Blood glucose, triglyceride, and cholesterol were measured over 60 days. Plasma TG clearance was determined via olive oil gavage. The effect of insulin on diabetic HTG was assessed on Day 60 post‐diabetes induction. Blood glucose increased over threefold, while plasma insulin decreased to 30% of controls after STZ injection in both wild type and heterozygous hamsters by Day 7, remaining stable for 60 days. Plasma TG in wild‐type hamsters remained unchanged at Day 7 post‐STZ injection but increased slightly thereafter. Conversely, heterozygous hamsters exhibited severe HTG by Day 7 until the end of the study. Olive oil gavage revealed much slower plasma triglyceride clearance in heterozygous hamsters compared to WT animals, despite significantly reduced lipoprotein lipase activity in post‐heparin plasma in both animals. Hyperglycemia and HTG in heterozygous hamsters were reversed to pre‐diabetic levels following intraperitoneal insulin administration. In conclusion, severe HTG in diabetic heterozygous LDL receptor deficient hamsters developed spontaneously and was insulin‐dependent. Thus, this hamster model holds promise for effectively studying the complications associated with human diabetic HTG.

## Graphical abstract

Heterozygous low density lipoprotein receptor hamsters treated with STZ developed diabetic spontaneous hypertriglyceridemia, providing a useful model to study human diabetes complicated with hyperlipidemia.

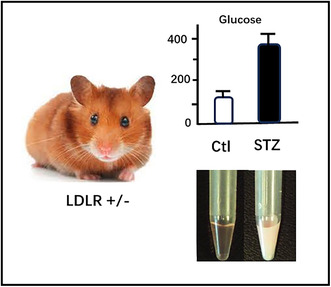

## 1 INTRODUCTION

Dyslipidemia is often associated with the development of diabetes and contributes to diabetic vascular complications.^1^ There are various forms of dyslipidemia in diabetes, of which hypertriglyceridemia (HTG) is the most common, especially in uncontrolled type I diabetic patients.^2^ The mechanisms of HTG in diabetes are not fully understood, but it is suggested that low expression of lipoprotein lipase (LPL), the critical enzyme involved in the hydrolysis of TG carried in plasma very low density lipoprotein (VLDL) and chylomicron, is responsible for the development of diabetic HTG.^3^

Destruction of pancreatic beta cells by different means can cause hyperglycemia in various animals such as mice, rats, hamsters and rabbits. Unlike humans, these diabetic animal models usually do not develop overt HTG unless they are challenged with a high fat diet.^4^ Even in genetic manipulated mouse models with deletion of genes involved in lipid metabolism, such as LDLR or ApoE, the diabetes induced by beta cell destruction only exhibited mild or moderate HTG if further dietary manipulations were not applied.^5^ This is because significant differences exist between mice and humans in lipid metabolism. For example, CETP and hepatic ApoB editing are absent in mice but present in humans.^6^

Hence, results from LDLR and ApoE knockout mice used as models of diabetic dyslipidemia are insufficient and more appropriate animal models for diabetic dyslipidemia research are needed. Lipid metabolism in Syrian Golden hamsters (referred to as hamsters hereafter) is similar to that in humans, and these hamsters have been used widely as human‐like models in studies of dyslipidemia and related diseases.^7^ However, because until now there have been no genetically engineered hamster models, the application of hamsters in diabetic dyslipidemia research has been limited although some meaningful discoveries have been made by different investigators.

Recently, we successfully generated the first dyslipidemic hamster model using gene editing technology, the LDL receptor knockout hamster. This model closely resembles human familial hypercholesterolemic (FH) patients in terms of genetic inheritance and the development of coronary atherosclerotic lesions.^8^

In the current study, we used the heterozygous LDLR knockout hamsters to induce diabetes and investigate the changes in triglyceride metabolism. We also verified whether insulin was responsible for these changes in triglyceride metabolism.

## 2 METHODS

### 2.1 Animals

The present study was performed on wild type and heterozygous LDLR deficient male Syrian Golden hamsters aged 8–10 weeks with body weights around 100–120 g. These hamsters were bred from an LDLR ablated line generated by CRISPR/CAS9 as previously described.^8^ They had free access to standard rodent chow diet and water and were kept in a clean, temperature‐controlled facility, under a 12‐h light–dark cycle. The experimental procedures were handled according to the *Guidelines of the Laboratory Animal Care* (NIH publication no. 85Y23, revised 1996) and approved by the Animal Care and Use Committee of the Peking University Health Science Center (LA2015‐012).

Hamsters were acclimated for 5 days before experiments. To produce insulin deficiency, hamsters were fasted overnight and given an IP injection of STZ (Sigma Chemical, St Louis, MO, USA), 60 mg/kg, dissolved in 50 mM citric acid buffer (pH 4.5), for three consecutive days. Seven days later, overnight‐fasted plasma glucose as well as TG and total cholesterol (TC) concentrations were measured. Eight to 10 animals per group were overnight‐fasted and anesthetized with isofluoran. Blood was collected from the retro‐orbital sinus and plasma was separated.

Insulin (0.75 U/kg body weight) was administered at the end of the 60‐day experiment to LDLR heterozygous hamsters (*n* = 10) with induced diabetes by intraperitoneal injections for three consecutive days.

### 2.2 Measurement of plasma lipids, glucose and insulin

Plasma TG and total cholesterol (TC) levels were measured with commercially available enzymatic kits (Wako Chemicals, Richmond, VA, USA), with cloudy and milky plasma samples diluted before measurement. Glucose concentration was measured with commercially available enzymatic kits (Sigma Chemical, St Louis, MO, USA). In order to eliminate the influence of turbidity caused by severe HTG, all plasma samples were ultracentrifugated with 50 000*g* for 20 min at 10°C to remove most TRLs before glucose measurement. Plasma insulin level was measured with commercial a. Rat/Mouse Insulin Elisa Kit for 96‐Well Plate (Linco Research, Cat. no. EZRMI‐13K), which was proven by us to have cross‐reaction with hamster plasma.

### 2.3 LPL activity in post‐heparin plasma

The protocol of post‐heparin plasma LPL activity assay was done according to Di Filippo et al.^9^ Briefly, blood was drawn 10 min after i.v. injection of 100 U/kg heparin, and the plasma was isolated at 4°C. LPL catalytic activity was measured using VLDL as substrate prepared by ultracentrifugation of human serum. The NEFAs (non esterified fatty acids) produced during incubation were assayed and LPL activity in post‐heparin plasma was expressed as mU/ml.

### 2.4 In vivo plasma TG clearance (oral fat load test)

After overnight fasting, heterozygous control and STZ‐treated hamsters were orally administered olive oil (10 μL/g) via gavage. Plasma samples were collected immediately (as the 0‐min sample) and at 30, 60, 120, 240 and 480 min post‐gavage. Plasma triglyceride concentrations were measured at all six time points, and a plasma triglyceride clearance curve was constructed for each animal.

### 2.5 Statistical analysis

Data were expressed as mean ± SEM. Comparison between two groups was performed by unpaired Student's *t*‐test (two‐tailed). *p* < 0.05 was considered statistically significant.

## 3 RESULTS

### 3.1 Induction of diabetes by STZ

**FIGURE 1:**
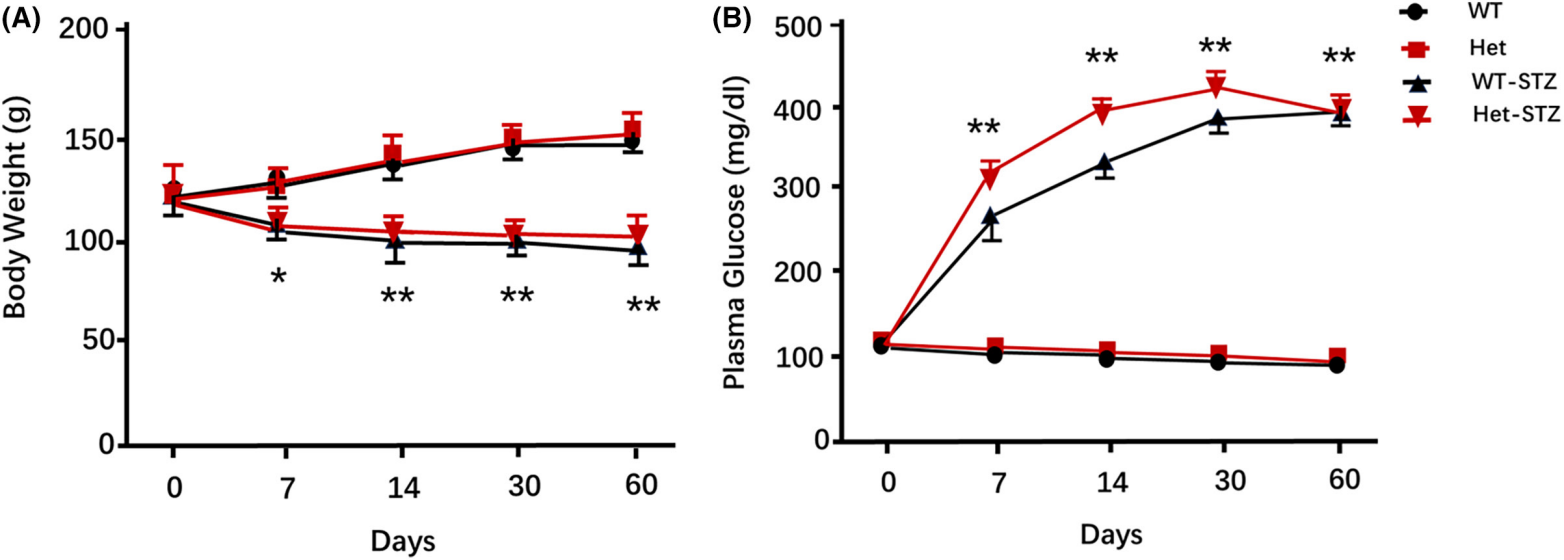
Change of body weight (A) and blood glucose (B) in WT and heterozygous hamsters after i.p. injection of STZ. WT and heterozygous hamsters (*n* = 8 for WT control group, *n* = 8 for Het. control group, *n* = 7 for WT‐STZ and *n* = 10 for Het‐STZ) demonstrated decreased body weight and increased blood glucose after i.p. injection of STZ over a 60 day period. *indicates *p* < 0.05 and **indicates *p* < 0.01.

Both WT and heterozygous hamsters developed diabetes within 2 week of i.p. injection of STZ at 60 mg/kg body weight in that their plasma glucose levels were higher than 300 mg/dL, while the glucose levels were about 100 mg/dL in respective control animals. At the same time, their body weights were reduced significantly compared to the controls (Figure 1A,B). The plasma insulin levels in diabetic hamsters were correspondingly reduced to nearly a third of that of non‐diabetic WT and heterozygous hamsters, as shown in Table 1. These results show clearly that type 1 diabetes can be induced in both WT and heterozygous hamsters by treatment with STZ.

**TABLE 1:** Reduced plasma insulin and LPL activity in post‐heparin plasma (PH‐LPL Act) in diabetic hamsters.

Groups	Insulin	PH‐LPL act	*n*	*p*
	Ng/mL	mU/mL		
WT	0.671 ± 0.123	112.5 ± 31.4	8	
Het	0.740 ± 0.216	132.8 ± 41.7	8	
WT‐STZ	0.205 ± 0.067	58.9 ± 22.4	7	<0.01
Het‐STZ	0.277 ± 0.084	47.3 ± 19.1	10	<0.05

*Note*: Compared with normal WT and heterozygous hamsters control hamsters the animals receiving ip injection of STZ showed significant reduction in plasma insulin and LPL activity at Day 60.

### 3.2 Development of spontaneous hypertriglyceridemia in diabetic heterozygous hamsters

**FIGURE 2:**
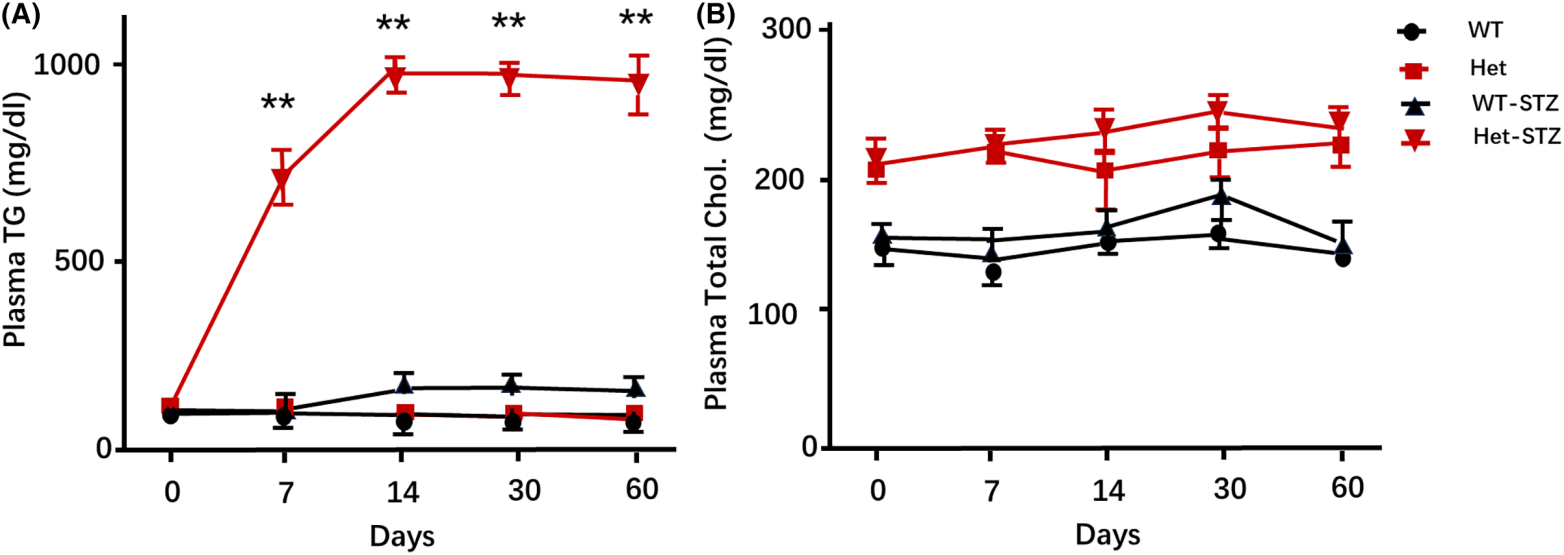
Change of plasma TG (A) and total cholesterol levels (B) in WT and heterozygous hamsters after treatment with STZ. After induction of diabetes with STZ, plasma triglyceride levels in LDLR heterozygous hamsters significantly increased as early as Day 7, reached a plateau by Day 14, and remained elevated until the end of the 60‐day experiment. In contrast, plasma triglyceride levels in WT hamsters showed only a slight increase after STZ administration. There were no significant changes in plasma total cholesterol levels in both wild‐type and heterozygous hamsters after STZ treatment (*n* = 8 for WT control group, *n* = 8 for Het. control group, *n* = 7 for WT‐STZ and *n* = 10 for Het‐STZ). *indicates *p* < 0.05 and **indicates *p* < 0.01.

**FIGURE 3:**
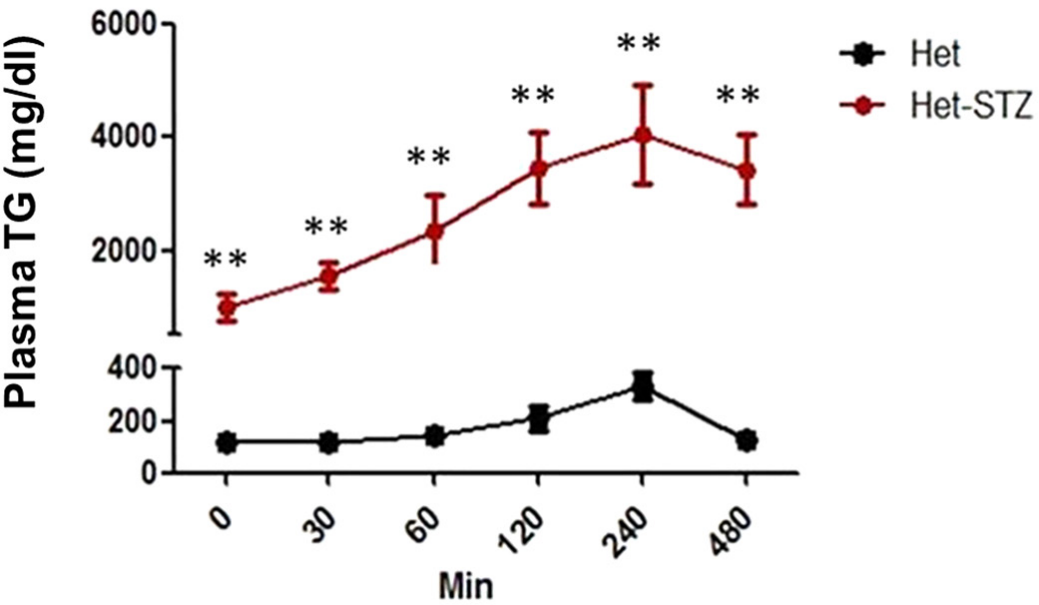
Slow plasma TG clearance after oral fat load in diabetic heterozygous hamsters. At Day 30 during the experiment, the oral fat tolerance test with olive oil gavage was performed. It was observed that the plasma triglyceride clearance rate was significantly reduced in the STZ‐induced diabetic hamsters (*n* = 10) compared to the control heterozygous hamsters (*n* = 8). *indicates *p* < 0.05 and **indicates *p* < 0.01.

After STZ injection, plasma TG in heterozygous hamsters on a chow diet had increased almost 7 times at Day 7 (126 ± 22 vs. 726 ± 134 mg/dL, *p* < 0.01, Figure 2A) but remained unchanged in the other three groups. At Day 14, the TG levels reached a plateau at around 1000 mg/dL, remaining at this level until Day 60. However, the TG levels in WT diabetic hamsters did not increase significantly over the course of 60 days on aa regular chow diet. Blood cholesterol levels were unchanged in both WT and heterozygous diabetic hamsters, although the heterozygous animals demonstrated higher levels of plasma cholesterol than the WT controls before and after STZ treatment (Figures 2B and 3). Since plasma TG is mainly hydrolyzed by LPL located in the vascular endothelium we determined LPL activity at this site which can be released by injection of heparin. As expected, the post‐heparin plasma LPL activity reduced significantly in both STZ‐treated WT and heterozygous hamsters, with more reduction in heterozygous animals (58.9 ± 22.4 and 47.3 ± 19.1 mU/mL in comparison to 112.5 ± 31.4 and 132.8 ± 41.7 mU in respective non‐diabetic controls, *p* < 0.01).

### 3.3 Retarded oral fat clearance in diabetic heterozygous hamsters

When oral fat load was assessed in diabetic and control heterozygous hamsters a huge difference was revealed. Although plasma TG in both groups reached its peak value at 240 min after oral fat load the TG level of diabetic hamsters was over 10 times higher than that of non‐diabetic controls (4010 ± 788 vs. 321 ± 34 mg/dL, *p* < 0.01). In non‐diabetic hamsters the plasma TG returned to the levels before oral fat load at 480 min but the TG of diabetic hamsters remained near the peak value at the same time point.

### 3.4 Reversal of HTG by administration of insulin

**FIGURE 4:**
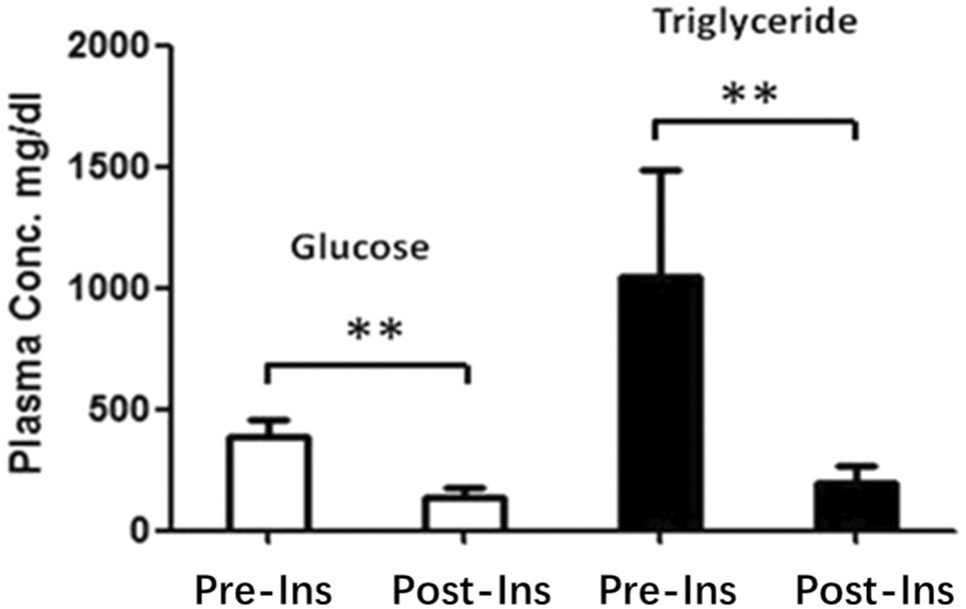
Reversal of HTG by administration of insulin. At the end of the 60‐day experiment, LDLR heterozygous hamsters (*n* = 10) with induced diabetes were given intraperitoneal insulin injections for three consecutive days. This treatment resulted in significant reductions in plasma glucose and triglyceride levels, which indicates that the hyperglycemia and hypertriglyceridemia in this model are both insulin dependent. **indicates *p* < 0.01.

To assess whether HTG in diabetic heterozygous hamsters was due to reduced insulin we administered insulin at the end of the experiment to diabetic hamsters by daily i.p. injection at a dose of 0.75 U/kg BW for three consecutive days. As shown in Figure 4, both plasma glucose and TG levels in diabetic heterozygous hamsters were reduced, to 30% and 20% of the levels pre‐insulin injection, respectively. This indicates clearly that hyperglycemia and HTG in these animals are both insulin dependent.

## 4 DISCUSSION

In the present study, we found that plasma TG levels in LDLR heterozygous hamsters increased about 7 times (as high as 1000 mg/dL) within a week of STZ injection and remained at that level until the end of the experiment, a more obvious increase than the increase of blood glucose. The results of the oral fat tolerance experiment showed that exogenous TG clearance of diabetic LDLR heterozygous hamsters was slowed down, which was related to the decrease of LPL activity in post‐heparin plasma. Further experiments proved that this severe HTG was insulin dependent and was similar to the development of hyperglycemia because the plasma TG quickly returned to almost normal levels after insulin injection. This is consistent with the clinical findings in type I diabetes patients whose plasma TG was basically normal as long as insulin was given adequately to control blood glucose.^10^ The molecular mechanisms underlying how the lack of insulin in humans leads to HTG involve several interconnected pathways in lipid metabolism. (a) Decreased LPL activity: insulin promotes the activation of LPL. In the absence of insulin, LPL activity is reduced, leading to impaired clearance of TG‐rich lipoproteins from the circulation.^11^ (b) Increased hepatic lipogenesis: insulin normally suppresses hepatic lipogenesis. If this suppression is reduced, hepatic production of TG‐rich VLDL particles will be increased. (c) Enhanced hormone‐sensitive lipase (HSL) activity: insulin normally inhibits HSL, the enzyme involved in the breakdown of stored triglycerides in adipose tissue. In the absence of insulin, HSL activity is increased, leading to increased release of free fatty acids from adipose tissue into circulation, which can be re‐esterified into triglycerides in the liver.^12^ (d) Altered expression of transcription factors: insulin regulates the expression of key transcription factors involved in lipid metabolism, such as sterol regulatory element‐binding proteins and peroxisome proliferator‐activated receptors. Insulin deficiency disrupts the balance of these transcription factors, resulting in dysregulation of lipid synthesis, storage, and metabolism.^13^ These mechanisms collectively contribute to the development of HTG in insulin deficiency in humans.

Our previous comparative study^14^ showed that high‐fat diet (HFD) containing 10% lard and 0.5% cholesterol for 2 weeks did not induce HTG in mice, and plasma TG even decreased. However, HTG was induced in hamsters (TG increased nearly 10 times) with the same HFD. After induction of diabetes mellitus by STZ, the plasma triglyceride levels of mice remained unchanged but were increased nearly three times in the hamsters. In mice with both HFD and diabetes, the TG levels were about two times greater than those in mice with diabetes alone. However, severe HTG developed in diabetic hamsters on HFD. Others using ApoE and LDLR knockout mice to study diabetes^5^ found that there was no obvious HTG in STZ‐induced diabetes mellitus in these mice. If these dyslipidemic diabetic mice were on HFD, then moderate to severe HTG could occur.^5^ We therefore thought that induction of diabetes in hamsters with similar gene‐deletions might result in HTG directly without HFD feeding, based on observations that either HFD or diabetes alone could induce moderate HTG in WT hamsters.

Indeed, the LDLR knockout hamster model created by our group demonstrated high plasma total cholesterol and LDL‐C not only in homozygous hamsters but also in heterozygous hamsters.^15^ Moreover, the hypercholesterolemia and HTG in heterozygotes were more severe than those of wild type hamsters after HFD feeding. Unlike LDLR knockout mice, LDLR knockout hamsters are like humans with a form of dominant HTG inheritance. Hence, we speculated that HTG would occur spontaneously if diabetes was induced in heterozygous LDLR deficient hamsters. The results of this study confirm our hypothesis and that we have successfully generated a unique animal model of spontaneous HTG in diabetic hamsters for the first time. Using this model, we demonstrated that severe HTG could be induced not only by HFD feeding but also by diabetes in these heterozygous LDLR deficient hamsters. However, it is not certain whether spontaneous HTG can be induced with type 2 diabetes in this hamster model since the majority of diabetes in humans is type 2 diabetes. This will be our future research goal.

Currently, the relationship between heterozygous familial hypercholesterolemia (FH) and diabetes is not clear. Because both diseases have a high incidence (the former accounts for 1/200–500 and the latter nearly reaches 1% in the population), it is therefore highly likely that diabetes occurs with FH. Paquette et al reported that the risk of developing coronary heart disease increased nearly three times in diabetic FH patients and identified HTG as one of concomitant risk factors.^16^ Hence, the severe HTG in our diabetic LDLR heterozygous hamsters could serve as an invaluable model of diabetic FH for elucidating the role of HTG in atherogenesis in these patients. Although spontaneous HTG developed in our diabetic LDLR heterozygous hamsters the potential mechanism(s) operating under this phenotype are yet to be explored. One plausible explanation could be the role of LDLR in the hepatic uptake of ApoB containing particles because ApoB is also one of the major protein components in TG‐rich VLDL/chylomicron. When LDLR function is impaired, the clearance of LDL will be slowed down. At the same time, the clearance of VLDL/chylomicron will be also affected but this may not be so apparent. The downregulation of LPL with a lack of insulin, as in the diabetic state, will result in the accumulation of VLDL/chylomicron and their remnants in the plasma. That happens in humans since HTG is one of the commonly seen complications in both type 1 and 2 diabetes. Lack of such phenotype in diabetic mouse models confirms that mice are not a suitable model for studying diabetic dyslipidemia. Hence, our study verifies the value of applying hamster models in this type of research.

In conclusion, we demonstrate that heterozygous LDL receptor deficient hamsters developed spontaneously sever HTG on normal chow diet when diabetes is induced by STZ and both hyperglycemia and HTG in diabetic hamsters are insulin dependent. These diabetic LDL receptor deficient hamsters can be used as a model for diabetic severe HTG for the study of vascular complications of human diabetic HTG.

## AUTHOR CONTRIBUTIONS

**George Liu:** Conceptualization; funding acquisition; project administration; resources; supervision; validation; writing – review and editing. **Ya‐Hong Ma:** Conceptualization; data curation; investigation; resources; writing – original draft. **Yuhui Wang:** Data curation; funding acquisition; methodology; project administration; resources; supervision; validation.

## FUNDING INFORMATION

National Natural Science Foundation of China (NSFC) 31520103909 and. 91739105 to G. Liu, 81770449 to Y. Wang.

## CONFLICT OF INTEREST STATEMENT

The authors declared no competing interests.

## ETHICS STATEMENT

All hamsters were housed in a temperature and humidity‐controlled room with a 12/12h light–dark cycle. All animal experiments were approved by the Animal Care and Use Committee of Peking University Health Science Center (LA2015‐012).
